# Rational Choices for Allocating Antiretrovirals in Africa: Treatment Equity, Epidemiological Efficiency, and Feasibility

**DOI:** 10.1371/journal.pmed.0030160

**Published:** 2006-03-28

**Authors:** David P Wilson, Sally M Blower

**Affiliations:** **1**University of New South WalesSydney, New South WalesAustralia; **2**University of California Los AngelesLos Angeles, CaliforniaUnited States of America

We agree with the thesis of Rosen et al. [ 1] that, despite initiatives such as the World Health Organization's “3 by 5” program, rationing of HIV/AIDS antiretroviral therapy (ART) will be necessary in the majority of African countries. Difficult choices will need to be made, and choices will be constrained due to the limited health infrastructure and lack of qualified health personnel in many African countries. Rosen et al. outlined a number of useful rationing systems and selection criteria [ 1].

However, some of the strategies they suggest are unfortunately not feasible in practice. For example, targeting behavioral core groups (high-risk groups, e.g., female sex workers) may well be impossible as it is not always possible to identify behavioral core groups. Furthermore, once the prevalence of HIV becomes extremely high in the general population (as it already is in many African countries), the concept of a behavioral core group will be relatively meaningless.

Previously, it has been shown, by using mathematical modeling, that targeting a virologic core group for treatment could be a very effective public health strategy for controlling herpes epidemics [ 2]. Only relatively few of the individuals infected with herpes simplex virus type 2 (HSV-2) are high viral shedders, and these individuals constitute the virologic core group. These individuals disproportionately contribute to the HSV-2 incidence rate. Thus, treating only the relatively few individuals who constitute the virologic core group has been shown to have a substantial effect on reducing the incidence of herpes [ 2]. Such a public health strategy for controlling herpes epidemics would be feasible, as it would be possible to identify the high viral shedders (i.e., the virologic core group) [ 2]. Such a strategy would also ensure that relatively few drugs would be needed to achieve epidemic control.

We suggest that when considering how to ration antiretrovirals among individuals with HIV in Africa, instead of targeting behavioral core groups, HIV virologic core groups should be targeted. Individuals with HIV who constitute the HIV virologic core group would be easy to identify simply by measuring viral load. The virologic core group will be composed of individuals with a high viral load. These individuals would not only be people in the late stage of disease, but would also include recently infected individuals, who have a high viral set point. Targeting the HIV virological core group would have several advantages: it would increase treatment equity (as these individuals have the greatest need for treatment), it would be epidemiologically efficient, and it would also be feasible. Whereas targeting the HIV behavioral core group would decrease treatment equity, it may or may not be epidemiologically efficient and would not be feasible.

Treatment equity and epidemiological efficiency are likely to have very different weights in each African society. Previously, we have shown, by using operational research methodology, that it is possible to use mathematics to decide how to achieve treatment equity [ 3]. Thus, it is possible to devise a mathematically ethical solution to decide how to allocate a scarce supply of antiretrovirals if the objective is to achieve treatment equity [ 3]. We have shown that treatment equity is only possible in some areas of South Africa if each of the available health-care facilities treat individuals with HIV in a large catchment area (radius of approximately 40–60 km ^2^) [ 3]. Hence, in some African countries, it may be impossible to achieve treatment equity even if it is possible to achieve a rationing strategy that would ensure the maximum reduction of the epidemic. Therefore, government officials and health policy experts in each African country will have to decide the relative weight that they wish to place on treatment equity versus epidemiological efficiency when they decide how to ration their scarce supply of antiretrovirals. We also stress that when using mathematical models to evaluate any rationing strategy, single scenarios should not be used to make complex decisions. There is a large degree of variability in the parameters that define each strategy and a great deal of heterogeneity in how a given strategy will be implemented. Accordingly, we recommend that time-dependent uncertainty boundaries should always be presented in any analysis when modeling is being used for health policy decision making [ 4]. In addition, detailed time-sensitivity analyses should also be presented so that it is possible to evaluate the robustness of the results [ 4].

Finally, we would like to stress the tremendous value in preferentially making ART available to mothers with HIV (especially women who are pregnant or breast-feeding), both to prevent vertical transmission and to act as a therapeutic intervention for the mother. Not only would this rationing strategy reduce the burden of orphan support, but the treatment regimen is relatively cheap and is extremely effective in reducing transmission to infants and increasing the life expectancy of the mother. Therefore, we strongly recommend that no pregnant woman with HIV be overlooked in the rationing of ART.
